# Continuance intention to use mobile learning among college students: integrating quality factors and the expectation confirmation model

**DOI:** 10.1038/s41598-026-35949-w

**Published:** 2026-01-19

**Authors:** Xin Qiu, Jianping Wu, Huixian Li

**Affiliations:** 1 School of Literature, Jiangxi Science & Technology Normal University, Nanchang, Jiangxi China; 2 School of Science, Hunan University of Science and Engineering, Yongzhou, Hunan China; 3 Basic Teaching Department, Hebei Vocational University of Industry and Technology, Shijiazhuang, Hebei China

**Keywords:** Continuance intention, Mobile learning, Quality, Expectation confirmation model, Psychology, Human behaviour

## Abstract

In light of the exponential rise of mobile device usage and advancements in information technology, mobile learning has ascended as an integral conduit for information and knowledge acquisition among educators and learners. Based on the expectation confirmation model, this study examines the mechanisms by which quality factors influence the continuance intention toward mobile learning among university students. A random sampling methodology was employed, yielding a robust sample of 278 students across three educational institutions in Jiangxi Province. A Partial Least Squares Structural Equation Modeling approach was used to analyze the data. The outcomes manifested significant positive associations across all relationships, with the exceptions being between system quality and perceived usefulness, information quality and confirmation, and service quality and perceived usefulness. Additionally, the study found no gender differences in the correlations between perceived usefulness and continuance intention or satisfaction and continuance intention. Based on these findings, the authors present suggestions for educational institutions, educators, system designers, and researchers to enhance the continuance intention to use mobile learning among college students.

**Supplementary Information:**

The online version contains supplementary material available at 10.1038/s41598-026-35949-w.

## Introduction

The rapid proliferation of mobile devices and continuous advancements in information technology have fundamentally reshaped the landscape of higher education. Mobile learning, which enables learners to access educational resources and engage in learning activities at any time and from any location, has become an integral component of contemporary university teaching and learning practices^1^. Owing to its flexibility, portability, and convenience, mobile learning is increasingly adopted by university students across diverse disciplines^2^. While prior studies have demonstrated the positive effects of mobile learning on learning efficiency, motivation, and satisfaction, the sustained use of mobile learning systems remains a critical challenge for higher education institutions.

Unlike initial adoption, students’ continuance intention (CI) reflects their long-term evaluation of a learning system after actual use and thus serves as a more reliable indicator of the success and sustainability of mobile learning initiatives^3^. Understanding the factors that drive university students to continue using mobile learning systems is therefore essential for system designers, educators, and policymakers seeking to enhance learning experiences and maximize the educational value of digital technologies. Despite growing scholarly attention to mobile learning, empirical evidence regarding the determinants of CI remains fragmented and context-dependent.

Existing research has employed a variety of theoretical frameworks to explain CI in technology-mediated learning environments, such as the Technology Acceptance Model (TAM)^4^, the Information Systems Success Model (ISSM)^5^ and the Expectation Confirmation Model (ECM)^5^. Among these, the ECM has been widely recognized for its explanatory power in post-adoption contexts. However, many previous studies have examined CI either from a user-centered perspective (e.g., perceived usefulness (PU), satisfaction (SAT))^6^, or from a system-centered perspective (e.g., system quality (SYQ), information quality (INQ), service quality (SEQ))^7^, with limited efforts to integrate these perspectives within a unified analytical framework. As a result, the joint effects of quality-related factors and post-adoption psychological mechanisms on mobile learning CI remain insufficiently understood.

To address this gap, the present study proposes an integrated research model that combines the ECM with key quality factors of mobile learning systems, including SYQ, INQ, and SEQ. By doing so, this study aims to provide a more comprehensive explanation of how institutional-level quality factors and individual-level cognitive and affective evaluations jointly influence university students’ CI to use mobile learning. In addition, given the mixed findings in prior research regarding gender differences in technology use, this study further explores whether gender moderates the relationships between PU, SAT, and CI.

This study makes three main contributions to the literature on mobile learning CI. First, it integrates quality factors (SYQ, INQ, and SEQ) into the ECM as contextual antecedents, offering a more comprehensive post-adoption explanation of CI. Second, it reveals the differentiated effects of quality dimensions, showing that SYQ and SEQ influence expectation confirmation, while INQ directly affects PU, thereby enriching existing ECM-based research. Third, by combining partial least squares structural equation modeling (PLS-SEM) with out-of-sample predictive assessment and rigorous multi-group analysis, this study strengthens both the methodological rigor and practical relevance of CI research.

The remainder of this paper is organized as follows. Section “Literature review and theoretical framework” reviews the relevant literature and presents the theoretical framework and hypotheses. Section “Hypotheses” describes the research methodology. Section “Results” reports the data analysis and results. Section “Discussion” presents the study’s findings and implications. Finally, section “Conclusions” concludes the paper, outlining its limitations and providing directions for future research.

## Literature review and theoretical framework

### Continuance intention

CI refers to an individual’s willingness to continue using a technology or system after initial adoption and actual usage experience^8^. Compared with initial adoption intention, CI captures users’ post-adoption evaluations and thus provides a more accurate indicator of the long-term success and sustainability of information systems^8^. In educational technology research, CI has been widely regarded as a critical outcome variable, as sustained use is essential for realizing the pedagogical value of digital learning systems^9,10^.

Early studies on CI in information systems were grounded mainly in expectation-based theories^11^. Among them, the ECM has been particularly influential in explaining users’ post-adoption behavior. ECM posits that CI is primarily determined by users’ SAT and PU, which are shaped by the confirmation of prior expectations through actual system use^3^. Extensive empirical evidence across diverse technological contexts—such as learning management systems^12^, e-learning platforms^10^, mobile applications^13^, and online services^14^—has consistently supported the robustness of this framework in explaining CI.

In the context of technology-enhanced learning, prior research has demonstrated that CI is influenced by a combination of cognitive evaluations and affective responses^6,15^. PU has been identified as a key cognitive determinant, reflecting learners’ beliefs about the extent to which a system enhances learning performance or efficiency. SAT, as an affective response, captures learners’ overall evaluation of their learning experience and has been shown to exert a direct and substantial impact on CI. Beyond individual-level perceptions, recent studies have increasingly emphasized the role of system-related and institutional factors in shaping CI^16^. Drawing on the ISSM, scholars have highlighted the importance of INQ, SYQ, and SEQ as critical antecedents that indirectly influence CI through users’ cognitive and affective evaluations^17^. High-quality learning content, reliable and user-friendly system design, and responsive technical support can enhance learners’ confirmation of expectations, PU, and SAT, thereby fostering sustained usage behavior. This line of research suggests that CI should be understood as the outcome of interactions between individual perceptions and system-level characteristics rather than as a purely user-driven decision.

In mobile learning research, CI has attracted growing scholarly attention due to the voluntary and self-directed nature of mobile technology use. Unlike mandatory institutional systems, mobile learning applications often compete with alternative learning tools and non-academic digital platforms, making sustained use particularly challenging. Empirical studies in mobile learning contexts have shown that CI is shaped not only by functional benefits but also by learners’ overall experiences, including convenience, flexibility, and perceived learning value^18^. However, existing findings remain fragmented, with some studies emphasizing user-centered factors and others focusing primarily on quality-related attributes of mobile learning systems.

Moreover, the role of individual differences, such as gender, in shaping CI remains inconclusive. While some studies report significant gender differences in the relationships between PU, SAT, and CI, others find no meaningful moderating effects^19^. These mixed findings suggest that the influence of gender may be context-dependent and warrant further empirical examination, particularly in mobile learning environments where usage patterns and expectations may differ across learner groups.

Overall, the existing literature underscores the multifaceted nature of CI and highlights the need for integrative frameworks that simultaneously consider post-adoption psychological mechanisms and system-level quality factors. Building on these insights, the present study situates CI within an integrated framework that combines the ECM with key quality dimensions of mobile learning systems, aiming to provide a more comprehensive understanding of university students’ sustained engagement with mobile learning.

### Expectation confirmation model

This study adopts the ECM as its sole theoretical framework to explain university students’ CI toward mobile learning. ECM was initially proposed by Bhattacherjee^20^ as an extension of the Expectation Confirmation Theory (ECT)^23^ and has been widely applied to examine users’ post-adoption behavior in information systems and technology-enhanced learning contexts.

Unlike pre-adoption models that focus on initial acceptance, ECM emphasizes users’ post-use cognitive evaluation and affective responses, making it particularly suitable for investigating CI, which refers to users’ willingness to persist in using a system after initial adoption. According to ECM, CI is primarily determined by three core constructs: CON, PU, and SAT. These constructs form a sequential causal mechanism in which users’ post-use evaluations shape their beliefs and emotions, which in turn influence their intention to continue using a system^20^.

Specifically, CON reflects the extent to which users’ experience with a system meets or exceeds their initial expectations. When users perceive that their expectations have been confirmed, they are more likely to evaluate the system as applicable and experience higher levels of SAT. PU represents users’ belief that using the system enhances their performance or learning effectiveness, while SAT captures users’ overall affective evaluation resulting from their usage experience^20^. Both PU and SAT are regarded as direct predictors of CI within the ECM framework.

Extensive prior research has demonstrated the robustness of ECM in explaining CI across diverse learning technologies, including learning management systems^21^, MOOCs^22^, and mobile learning environments^24^. These studies consistently indicate that ECM provides a strong theoretical foundation for understanding how learners’ post-use evaluations translate into sustained usage behavior.

### Extension of ECM in the mobile learning context

Although ECM offers a parsimonious and powerful explanation of CI, scholars have noted that users’ CON and subsequent evaluations are shaped by their concrete usage experiences within specific technological contexts. In mobile learning environments, learners’ post-use evaluations are not formed in isolation but are influenced by their perceptions of how well the system performs during actual use^25^.

Within the ECM perspective, such performance-related perceptions can be understood as antecedent conditions that affect users’ CON of expectations, PU, and SAT. In mobile learning, students interact continuously with learning content, system interfaces, and support services. Their evaluations of these experiences play a critical role in shaping whether initial expectations are confirmed and whether the system is perceived as beneficial and satisfying. Accordingly, this study incorporates INQ, SYQ, and SEQ as context-specific antecedent factors within the ECM framework. These constructs are not treated as components of an additional theoretical model, but rather as experiential cues that influence users’ post-adoption evaluations as conceptualized by ECM. INQ reflects students’ perceptions of the accuracy, usefulness, clarity, and relevance of learning content delivered through mobile learning platforms^26^. SYQ refers to students’ perceptions of the functionality, usability, stability, and ease of navigation of mobile learning systems^26^. SEQ captures students’ perceptions of the support and assistance provided during mobile learning, including responsiveness to problems and the availability of help^26^. From an ECM standpoint, these quality-related perceptions shape how learners assess their post-use experience and thus influence CON, PU, and SAT, ultimately affecting CI.

## Hypotheses

According to the ISSM proposed by William and Ephraim^27^, INQ, SYQ, and SEQ are crucial determinants of user behavior in relation to information systems. Drawing from the ECM, CON is defined as the user’s perception of the benefits they derive from their actual usage experience relative to their initial expectations of the IT services/products^20^. Past research has investigated the correlation between the quality of IT services/products and CON^28,27,30^. For instance, Cheng^28^ treated SYQ, INQ, and SEQ as antecedents of users’ CI in utilizing e-learning systems. The study deduced that the quality factor of an IS influences the CON of user expectations^30^. In addition, cumulative evidence from meta-analytic and integrative reviews further supports the role of SYQ, INQ, and SEQ as key antecedents of users’ post-adoption evaluations. Meta-analytic findings indicate that quality perceptions consistently shape users’ CON of expectations and subsequent beliefs across information system contexts^31^. Empirical evidence suggests that when users perceive the quality of an e-learning system as surpassing their expectations, they respond favorably to the e-learning system. Hence, this study concludes that high-level platform qualities, encompassing INQ, SYQ, and SEQ, offered by mobile learning, positively impact expectation CON. Based on this, we proposed the following hypotheses:

**H1**: INQ has a significant positive influence on CON.

**H2**: SYQ has a significant positive influence on CON.

**H3**: SEQ has a significant positive influence on CON.

Past research has explored the correlation between the quality of IT services/products, namely INQ, SYQ, SEQ, and PU^32,31,32,35^. For instance, Al-Fraihat et al.^32^ developed and empirically tested a comprehensive model through PLS-SEM to evaluate e-learning systems. The findings indicated that SEQ, INQ, SYQ, and other constructs were key drivers of PU, accounting for 54.2% of the variance in PU. Similarly, Wibowo and Sari^35^, building on DeLone and McLean’s information system model and the technology-organization-environment framework, employed PLS-SEM to identify the critical factors determining the success of Enterprise Resource Planning systems. The study concluded that SYQ, INQ, and SEQ have a significant influence on PU. In addition to prior empirical findings, meta-analytic evidence has demonstrated that INQ, SYQ, and SEQ exert robust effects on PU and other belief-related constructs in post-adoption contexts^36^. This study posits that the greater the SYQ, SEQ, and INQ of a mobile learning experience, the higher its PU among students. Consequently, based on the results of the previous studies, the hypothesis in this study is as follows:

**H4**: INQ has a significant positive influence on PU.

**H5**: SYQ has a significant positive influence on PU.

**H6**: SEQ has a significant positive influence on PU.

Prior research has explored the relationship between CON and PU^37,38^^39,40^. For instance, Muñoz-Carril et al.^38^ conducted a study to identify the determinants of student SAT scores and the perceived impact on learning in the realm of Computer-Supported Collaborative Learning. By utilizing a sample of 701 students from a virtual university and implementing the PLS-SEM for data analysis, their findings highlighted CON as a significant predictor of PU. Likewise, Zhang et al.^7^ drew upon the ECT and IS success model as their theoretical framework in their exploration of key predictors of online learning system CI. Their study, based on a random selection of 537 undergraduates, demonstrated that CON exerts a significant influence on PU in online learning. In line with these findings, this study posits that the higher the level of positive CON from undergraduates towards mobile learning, the more they perceive the system as applicable in their learning process. Consequently, based on the results of the previous studies, the hypothesis in this study is as follows:

**H7**: CON has a significant positive influence on PU.

Several studies have sought to assess the influence of CON on SAT^38,37,38,43^. For instance, Muñoz-Carril et al.^38^, in their study aimed at identifying factors that impact student SAT within the domain of computer-supported collaborative learning, employed PLS-SEM for data analysis on a sample of 701 students from a virtual university. Their findings pointed to CON as a crucial precursor of SAT. Similarly, Dai et al.^42^ selected the ECM as their theoretical framework in their exploration of the factors underlying the intention to continue learning in massive open online courses. Data analysis through the PLS-SEM revealed a significant positive relationship between CON and SAT. Notably, a comprehensive meta-analysis of studies grounded in the ECM confirms that expectation CON has a substantial and statistically significant effect on SAT across diverse post-adoption settings^11^.In line with these studies, this research suggests that a more positive CON of expectations from mobile learning correlates with a higher level of user SAT. Consequently, based on the results of the previous studies, the hypothesis in this study is as follows:

**H8**: CON has a significant positive influence on SAT.

Several studies have endeavored to elucidate the influence of PU on SAT across various contexts^7,44,41,42,47^. For instance, Li and Phongsatha^44^ employed a PLS-SEM to evaluate a hypothesized model in the context of blended learning. The study was based on a survey conducted with 635 junior high school students, and the results underscored the critical impact of PU on the SAT. Similarly, Wang et al.^46^ validated factors affecting students’ online learning confidence in a comparative analysis of live video learning, pre-recorded video learning, and hybrid video learning during the COVID-19 pandemic. Their findings indicated that PU serves as a vital precursor to SAT. In alignment with these findings, this study suggests that undergraduate students who perceive that mobile learning enhances their academic performance or learning efficiency are likely to experience increased SAT. Consequently, based on the results of the previous studies, the hypothesis in this study is as follows:

**H9**: PU has a significant positive influence on SAT.

A growing body of recent research indicates that PU has a positive influence on CI^7,48,45,50^. Suriazdin et al.^48^, for example, found that users were inclined to persist in using MOOCs because they considered them beneficial for enhancing their academic achievements. Similarly, Yang et al.^50^ posited that PU is a crucial precursor to CI within the context of information system usage. In addition, this relationship is also supported by meta-analytic evidence showing that PU is a consistent predictor of affective evaluations, including SAT, across technology adoption and CI studies^51^. In light of these findings, this study proposes that when students perceive that mobile learning can improve their academic performance or learning efficiency, they will continue to use it. Consequently, based on the results of the previous studies, the hypothesis in this study is as follows:

**H10**: PU has a significant positive influence on CI.

An extensive body of existing literature has substantiated the relationship between SAT and CI, concluding that user SAT significantly influences their intention to reuse a learning system^7,45,50,52^. For instance, Rekha et al.^45^ investigated learners’ CI, selecting a sample of 383 undergraduate and postgraduate students from India and conducting their analysis using PLS-SEM. The results indicated that SAT is a key predictor of CI. In addition, recent meta-analytic research synthesizing evidence from online and mobile learning contexts has confirmed that SAT is the strongest predictor of learners’ CI, providing robust cumulative support for these relationships^10^. Consistent with these findings, this study proposes that the greater the users’ SAT with mobile learning, the stronger their intention to continue using it. Consequently, based on the results of the previous studies, the hypothesis in this study is as follows:

**H11**: SAT has a significant positive influence on CI.

In this research, gender plays a crucial role. Several scholars have explored how gender moderates the relationship among PU, SAT, and CI^49,53,50,51,56^. A segment of these studies suggests that PU, SAT, and CI exhibit significant differences across genders^54,56^. For instance, Nguyen et al.^56^ found that the linkage between PU and CI is significantly stronger in males than in females. Contrastingly, other research indicates no considerable differences in PU, SAT, and CI based on gender^55,56^. Specifically, Nguyen et al.^56^ utilized the UTAUT framework to scrutinize the moderating role of gender in their proposed model, and their findings revealed that gender did not significantly influence SAT and CI. Moreover, integrative and meta-analytic reviews suggest that gender differences in post-adoption relationships are often weak or inconsistent, particularly in voluntary technology use contexts, indicating that such effects may be highly context-dependent^57^. Consequently, based on the results of the previous studies, the hypothesis in this study is as follows:

**H12**: There are gender differences in the relationship between the PU of mobile learning and the CI.

**H13**: There are gender differences in the relationship between the SAT of mobile learning and the CI.

**Fig. 1:**
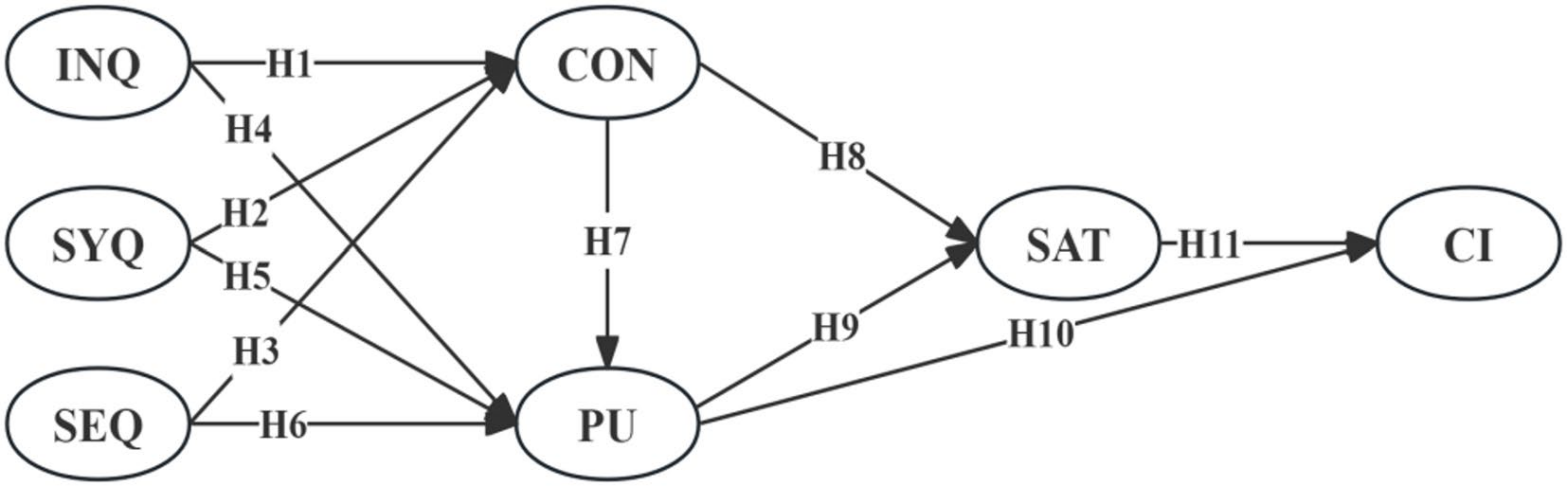
Hypothesized model.

According to the previous hypotheses, the conceptual model of this study is proposed (Fig. 1).

## Methods

### Participants

This study was approved by the Institutional Ethics Committee of the authors’ affiliated university. The study was conducted in strict accordance with the ethical guidelines for the protection of research participants (Ethics approval No. HVUIT-2022-09-0006). For this research, data were gathered from three Chinese universities using the Wenjuan Star platform (https://www.wjx.cn). The participants comprised students who had utilized mobile learning for a minimum of one semester. We employed a random sampling strategy to select participants. Before their participation, individuals were explicitly informed that the collected data would be exclusively used for scholarly research purposes. Emphasis was placed on the rigorous protection of their personal privacy. Based on this knowledge, their participation can be construed as giving informed consent. An online survey collected a total of 298 responses. To ensure that participants had authentic and sufficient experience with mobile learning, this study established explicit inclusion and exclusion criteria. The inclusion criteria were as follows: (1) participants had to be full-time undergraduate students enrolled at three universities in Jiangxi Province; (2) they were required to have used a mobile learning system continuously for at least one academic semester (approximately 3–4 months); (3) they participated voluntarily and provided informed consent online; (4) they were able to complete all key measurement items in the questionnaire. The exclusion criteria included: (1) students who had never used mobile learning or whose usage duration was shorter than one semester; (2) invalid questionnaires identified by abnormally short response times, uniform response patterns, or logical inconsistencies; (3) respondents who were not undergraduate students (e.g., preparatory students or postgraduates) or who were not enrolled in the target institutions specified in this study; (4) questionnaires that were incomplete or contained a large proportion of missing values. Based on these criteria, a total of 278 valid questionnaires were retained for subsequent analysis.

In the hypothesized model of this study, the maximum number of arrows pointing to any endogenous latent construct is three. According to Hair Jr et al.^58^, to achieve a model explanatory power of R2 = 0.10 at the 5% significance level, the minimum required sample size is 124. This study employed a sample of 278 participants, which substantially exceeds this minimum requirement, thereby ensuring the robustness and reliability of the research findings. We conducted chi-square tests to verify the representativeness of our sample, which consisted of 41.23% males and 53.14% females (*p* = 0.500). We found no statistically significant divergence between the sample and the population distribution, signifying the sample’s high representativeness. The data collection process spanned from February to May 2023. The demographic characteristics of participants are shown in Table 1.

**Table 1:** Demographic profile of the participants (*N* = 278).

Characteristics	Category	*N*	%
Gender	Male	151	54.3%
	Female	127	45.7%
Grade	Freshman	53	19.1%
	Sophomore	76	27.3%
	Junior	97	34.9%
	Senior	52	18.7%

### Measurements

The data collection instrument is divided into two sections. The initial section gathers the demographic data of the participants, which encapsulates aspects such as gender, age, grade, and subject of study. The subsequent section aims to appraise seven constructs derived from the theoretical framework. To elaborate, this portion includes 26 indicators (Table 2). A 7-point Likert scale was used, ranging from 1 (strongly disagree) to 7 (strongly agree). All the questions were formulated based on pertinent academic literature.

The students’ perspectives on the PU of mobile learning were evaluated utilizing a four-item scale designed by Mohammadi^59^. In alignment with the goals of the current study, “Moodle” was replaced with “mobile learning” in the original scale—for instance, “Using mobile learning enables me to accomplish my tasks more quickly.” Bhattacherjee^20^ three-item scale was employed to gauge students’ CON of mobile learning. The original scale underwent modification by substituting “OBD” with “mobile learning”—for example, “My experience with using mobile learning was better than what I expected.” Cheng^33^ scale was used to appraise the students’ CI with their mobile learning, a scale comprising four items, such as “I will continue to use mobile learning for further study in the future.” Almaiah and Alismaiel^60^ employed a three-item scale to assess participants’ SAT with mobile learning. For instance, “In general, I am satisfied with mobile learning.” Lastly, to assess INQ, SEQ, and SYQ, we used the scale developed by Urbach et al.^61^. Each construct is evaluated through four items.

**Table 2:** Constructs measurement and source.

Construct	Item	Measurement	Source
PU	PU1	Mobile learning can help me complete tasks faster	Mohammadi^59^
	PU2	Mobile learning can improve my academic performance	
	PU3	Mobile learning can help me study effectively	
	PU4	Overall, mobile learning is useful	
CON	CON1	The mobile learning experience was better than I expected	Bhattacherjee^20^
	CON2	The quality of service provided by mobile learning was better than I expected	
	CON3	Overall, most of what I expected from mobile learning was confirmed	
SAT	SAT1	My decision to use mobile learning was a good one	Almaiah and Alismaiel^60^
	SAT2	Mobile learning has fulfilled my expectations	
	SAT3	Overall, I’m satisfied with mobile learning	
CI	CI1	I will continue to use mobile learning for further study in the future	Cheng et al.^62^
	CI2	In the future, I will use mobile learning to study regularly	
	CI3	In the future, I will often use mobile learning for learning	
	CI4	I would prefer to continue using mobile learning rather than other alternatives	
SEQ	SEQ1	When I needed support during the use of mobile learning, the service staff was always very willing to help me	Urbach et al.^61^
	SEQ2	When I encountered problems while using mobile learning, the service staff always gave me individual attention	
	SEQ3	Service personnel provide mobile learning-related services at the agreed-upon time	
	SEQ4	The service staff had enough knowledge to answer my questions about mobile learning	
INQ	INQ1	The information provided by mobile learning is useful	Urbach et al.^61^
	INQ2	The information provided by mobile learning is understandable	
	INQ3	The information provided by mobile learning is interesting	
	INQ4	The information provided by mobile learning is reliable	
SYQ	SYQ1	Mobile learning is easy to navigate	Urbach et al.^61^
	SYQ2	In mobile learning, I can easily find the information I need	
	SYQ3	The mobile learning structure is well-designed	
	SYQ4	Mobile learning is easy to use	

### Statistical analysis

Data was analyzed using PLS-SEM 4.0. PLS-SEM has several advantages in data analysis, including the ability to handle non-normal data, small sample sizes, and the maximization of the explanatory power of variance in endogenous latent variables, as well as the ability to handle complex models^63^. The purpose of this study is to investigate the maximum explanatory power of CI variance by INQ, SEQ, SYQ, SAT, PU, and CON. The sample size is only 278 and includes seven constructs that belong to a complex model. Therefore, PLS-SEM is suitable for data analysis in this study.

Data analysis includes three aspects. First is the outer model test, namely, construct reliability and validity. Reliability encompasses item reliability, composite reliability, and Cronbach’s alpha (α). The validity includes convergent validity and discriminant validity. To validate the discriminant validity of the construct, the Fornell-Larcker criterion and cross-loadings were tested. Second, the inner model, including the collinearity test, significance test of model relations, model’s explanatory power (R^2^), effect size (f^2^), model’s predictive power (Q^2^), and common method variance (CMV). Finally, a multi-group analysis examined gender differences between mobile learning SAT, PU, and CI.

Although PLS-SEM does not require multivariate normality assumptions, descriptive statistics (i.e., mean, standard deviation, coefficient of variation, skewness, and kurtosis) were additionally reported to provide a comprehensive overview of the data distribution. These statistics are presented for descriptive purposes only and were not used as a prerequisite for model estimation.

## Results

### Descriptive statistics

Descriptive statistics for the primary constructs are presented in Table 3. The mean values of all constructs range from 3.808 to 3.917, indicating a moderate to relatively high level of agreement among respondents. The standard deviations range from 0.911 to 0.984, indicating an acceptable degree of variability in participants’ responses. Regarding data distribution characteristics, all constructs exhibit negative skewness values (ranging from − 1.136 to −0.834), indicating a tendency toward higher response categories. The kurtosis values range from 0.682 to 1.520, reflecting moderately peaked distributions. Overall, these descriptive statistics provide an informative overview of the data distribution and indicate no extreme deviations.

**Table 3:** Descriptive statistics.

Constructs	Mean	Standard deviations	Skewness	Kurtosis
PU	3.887	0.944	−1.136	1.520
CON	3.840	0.984	−0.872	0.838
SAT	3.870	0.911	−0.996	1.303
CI	3.808	0.966	−0.878	0.751
SEQ	3.831	0.965	−0.834	0.682
INQ	3.916	0.929	−1.051	1.419
SYQ	3.917	0.951	−1.067	1.356

### Outer model

The evaluation of the measurement model encompasses the analysis of both indicator reliability and internal consistency reliability, contributing to the overall assessment of the measures’ dependability. Additionally, the validity assessment focuses on examining convergent validity and discriminant validity.

The inaugural phase of the measurement model evaluation involves scrutinizing the outer loadings of the indicators. As stated by Hair Jr et al.^58^, the standardized outer loading of each indicator should be at a minimum of 0.708. The reliability of the construct was verified using Cronbach’s α and composite reliability, with values ranging between 0.7 and 0.9 deemed satisfactory^58^. For assessing the construct’s convergent validity, the Average Variance Extracted (AVE) should exceed 0.5, indicating a high correlation between the indicators and the construct^58^. Discriminant validity represents the degree to which a construct genuinely differs from other constructs. Historically, researchers have relied on the Fornell-Larcker criterion for assessing discriminant validity^64^. According to Fornell and Larcker^64^, the square root of each construct’s AVE should surpass its highest correlation with any other construct. Furthermore, if the cross-loading values of all items from one indicator outperform those of items from other indicators, it can be inferred that the model has achieved discriminant validity^65^. Tables 4 and 5, and 6 show that all the results meet the above criteria, indicating that the construct in this study has satisfactory reliability and validity.

**Table 4:** Reliability and validity.

Constructs	Items	loadings	Cronbach’s α	Composite reliability	AVE
CON	CON1	0.952	0.946	0.947	0.903
	CON2	0.95			
	CON3	0.95			
SAT	SAT1	0.953	0.952	0.952	0.912
	SAT2	0.952			
	SAT3	0.96			
CI	CI1	0.914	0.943	0.948	0.854
	CI2	0.937			
	CI3	0.965			
	CI4	0.881			
SEQ	SEQ1	0.941	0.958	0.959	0.889
	SEQ2	0.937			
	SEQ3	0.948			
	SEQ4	0.944			
INQ	INQ1	0.953	0.959	0.959	0.89
	INQ2	0.939			
	INQ3	0.938			
	INQ4	0.943			
SYQ	SYQ1	0.951	0.962	0.963	0.898
	SYQ2	0.958			
	SYQ3	0.95			
	SYQ4	0.931			
PU	PU1	0.932	0.959	0.959	0.891
	PU2	0.953			
	PU3	0.952			
	PU4	0.94			

**Table 5:** Fornell-Larcker criteria.

	CI	CON	INQ	PU	SAT	SEQ	SYQ
CI	**0.924**						
CON	0.887	**0.95**					
INQ	0.874	0.838	**0.943**				
PU	0.888	0.903	0.854	**0.944**			
SAT	0.903	0.931	0.87	0.887	**0.955**		
SEQ	0.889	0.843	0.903	0.825	0.865	**0.943**	
SYQ	0.863	0.856	0.931	0.84	0.885	0.892	0.947

Note: The diagonal bold data is the square root of AVE.

**Table 6:** Cross-loadings.

	CI	CON	INQ	PU	SAT	SEQ	SYQ
CON1	0.874	0.952	0.812	0.889	0.885	0.797	0.821
CON2	0.821	0.95	0.776	0.85	0.883	0.803	0.79
CON3	0.833	0.95	0.802	0.836	0.887	0.804	0.829
SAT1	0.862	0.891	0.83	0.868	0.953	0.825	0.844
SAT2	0.839	0.89	0.793	0.812	0.952	0.809	0.817
SAT3	0.887	0.886	0.869	0.861	0.96	0.842	0.872
CI1	0.914	0.862	0.828	0.875	0.893	0.822	0.822
CI2	0.937	0.814	0.818	0.834	0.833	0.81	0.799
CI3	0.965	0.849	0.833	0.841	0.857	0.852	0.817
CI4	0.881	0.745	0.747	0.719	0.744	0.803	0.747
SEQ1	0.882	0.836	0.84	0.806	0.843	0.941	0.863
SEQ2	0.84	0.785	0.823	0.792	0.802	0.937	0.821
SEQ3	0.814	0.778	0.852	0.742	0.805	0.948	0.824
SEQ4	0.813	0.776	0.89	0.768	0.808	0.944	0.854
INQ1	0.843	0.822	0.953	0.842	0.846	0.845	0.864
INQ2	0.806	0.769	0.939	0.793	0.803	0.839	0.885
INQ3	0.822	0.796	0.938	0.782	0.811	0.881	0.877
INQ4	0.826	0.775	0.943	0.803	0.823	0.842	0.889
SYQ1	0.843	0.815	0.902	0.817	0.853	0.89	0.951
SYQ2	0.844	0.845	0.883	0.813	0.856	0.868	0.958
SYQ3	0.788	0.786	0.892	0.771	0.826	0.822	0.95
SYQ4	0.793	0.796	0.852	0.781	0.816	0.797	0.931
PU1	0.832	0.842	0.821	0.932	0.829	0.758	0.806
PU2	0.849	0.839	0.811	0.953	0.825	0.801	0.78
PU3	0.844	0.865	0.801	0.952	0.831	0.788	0.785
PU4	0.827	0.864	0.791	0.94	0.865	0.768	0.801

### Inner model

#### Collinearity

To assess collinearity, the Variance inflation factor (VIF) values in the predictor constructs were adopted. According to Hair Jr et al.^58^, VIF values should be below five and ideally below three to ensure that collinearity has no substantial effect on the structural model estimates. Table 7 indicates that all VIF values were between 2.395 and 4.698, thus meeting the recommended level.

**Table 7:** VIF values of predictor constructs.

	CI	CON	INQ	PU	SAT	SEQ	SYQ
CI							
CON				4.258	3.425		
INQ		3.286		3.35			
PU	3.698				2.425		
SAT	4.698						
SEQ		3.056		3.563			
SYQ		2.395		2.195			

#### Significance of the structural model relationship

To answer question 1, the significance of the structural model relationship was assessed using the bootstrapping algorithm in PLS-SEM. According to Hair Jr et al.^58^, t-statistics (t > 1.96), p-values (*p* < 0.05), and confidence intervals (excluding zero) were used to test the significance of the relationship. Table 8 presents the path coefficient, confidence interval, T-statistics, and p-values. Among the direct predictors of CON, SYQ (β = 0.434, t = 3.433, *p* = 0.001) exerted the strongest effect, followed by SEQ (β = 0.345, t = 3.150, *p* = 0.002). Therefore, H2 and H3 were supported. However, INQ did not have a significant effect on CON (β = 0.123, t = 1.172, *p* = 0.241); thus, H1 was not supported. Similarly, INQ had a significant and positive influence on PU (β = 0.337, t = 3.155, *p* = 0.002), providing support for H4. In contrast, SYQ (β = −0.020, t = 0.156, *p* = 0.876) and SEQ (β = 0.004, t = 0.036, *p* = 0.971) did not significantly predict PU; therefore, H5 and H6 were not supported. CON had a significant positive effect on both PU (β = 0.635, t = 7.371, *p* < 0.001) and SAT (β = 0.702, t = 9.188, *p* < 0.001), thus confirming H7 and H8. Likewise, PU significantly and positively affected SAT (β = 0.253, t = 3.228, *p* = 0.001) and CI (β = 0.406, t = 6.523, *p* < 0.001), supporting H9 and H10. Finally, SAT exerted a significant positive influence on CI (β = 0.543, t = 8.62, *p* < 0.001), thereby supporting H11.

**Table 8:** Result of the significance of the structural model relationship.

Hypotheses	Relationship	β	2.50%	97.50%	t statistics	*P* values	Results
H1	INQ → CON	0.123	−0.079	0.337	1.172	0.241	Not supported
H2	SYQ → CON	0.434	0.191	0.688	3.433	0.001	Supported
H3	SEQ → CON	0.345	0.114	0.544	3.15	0.002	Supported
H4	INQ → PU	0.337	0.133	0.554	3.155	0.002	Supported
H5	SYQ → PU	−0.02	−0.29	0.204	0.156	0.876	Not supported
H6	SEQ → PU	0.004	−0.18	0.231	0.036	0.971	Not supported
H7	CON → PU	0.635	0.458	0.794	7.371	0.000	Supported
H8	CON → SAT	0.702	0.551	0.857	9.188	0.000	Supported
H9	PU → SAT	0.253	0.095	0.403	3.228	0.001	Supported
H10	PU → CI	0.406	0.29	0.537	6.523	0.000	Supported
H11	SAT → CI	0.543	0.41	0.657	8.62	0.000	Supported

#### Explanatory power

To address Research Question 2, this study examined the explanatory power of the model. The model’s explanatory power refers to its ability to fit the available data by assessing the strength of association represented by the PLS path model^66^. The coefficient of determination, also known as the R-squared value, is frequently used to assess the explanatory power of the structural model. The R^2^ value oscillates between 0 and 1, with superior values indicating enhanced explanatory power^58^. As posited by Urbach and Ahlemann^67^, values close to 0.67 are considered substantial, those around 0.33 are regarded as moderate, and those near 0.19 are considered weak. Table 9 illustrates that our model possesses significant explanatory power. Therefore, research question 2 has been addressed.

**Table 9:** Explanatory power.

Construct	*R* ^2^	Result
CI	0.851	Substantial
CON	0.765	Substantial
PU	0.847	Substantial
SAT	0.878	Substantial

#### Effect size

The f^2^ effect size represents the change in R-squared value when a specific predecessor construct is omitted from the model. Guidelines for assessing f^2^ are that values of 0.02, 0.15, and 0.35, respectively, represent small, medium, and significant effects of the exogenous latent variable^58^. According to Table 10, SEQ and SYQ have a small effect size on CON. Similarly, INQ has a small effect size of 0.079 on PU, and PU has a small effect size of 0.097 on SAT, too. Additionally, PU and SAT have a significant effect on CI. CON has a large effect size on PU and SAT. On the contrary, INQ does not affect CON (0.007). SEQ and SYQ do not affect PU either.

**Table 10:** Effect size.

	CI	CON	INQ	PU	SAT
CI					
CON				0.619	0.746
INQ		0.007		0.079	
PU	0.235				0.097
SAT	0.42				
SEQ		0.084		0.000	
SYQ		0.095		0.000	

#### Predictive power

To produce generalizable findings, it is necessary to test not only the model’s in-sample predictive power but also the out-of-sample predictive power^58^. The latter is the model’s predictive power. Currently, there are two indicators used to assess predictive power: Q^2^ and RMSE. Recently, some research has cast doubt on Q2 because this method combines the assessment of explanatory and predictive power^66,68^. Therefore, in this study, the RMSE method was used to evaluate the predictive power of the model. The smaller the RMSE value, the higher the predictive power of the model. According to Hair Jr et al.^58^, CI, comprising four indicators (Q15, Q16, Q17, Q18), is the key endogenous construct of the model. The RMSE method consists of two steps: interpreting the Q2predict values and comparing the RMAE values generated by the PLS-SEM analysis with those produced by the naïve linear regression model benchmark. In Table 11, all four indicators of CI have Q^2^_predict_ values larger than zero, suggesting that the PLS path model outperforms the most naïve benchmark. Then, comparing the RMSE values, the same number of indicators in PLS-SEM analysis yields smaller prediction errors compared to LM^58^. Therefore, this indicates the model has moderate predictive power.

**Table 11:** PLS_predict_ results Report.

Indicators	Q^2^_predict_	PLS-SEM_RMSE	PLS-SEM_MAE	LM_RMSE	LM_MAE
Q15	0.705	0.525	0.342	0.521	0.298
Q16	0.685	0.532	0.344	0.554	0.311
Q17	0.727	0.504	0.338	0.49	0.288
Q18	0.611	0.67	0.496	0.672	0.425

### Multi-group analysis

To answer research question 3, this study conducted a multi-group analysis based on the gender of the university students. Multi-group analysis provides an effective strategy for exploring heterogeneity across various data groups, thereby reducing the likelihood of misinterpreting findings^69^. Henseler et al.^70^ stated that multi-group analysis engages a three-tiered procedure to scrutinize the Measurement Invariance of Composite Models (MICOM) and to interpret permutation results. This procedure encompasses configural invariance, compositional invariance, and composite equality^70^. To establish configural invariance, in the first step, the following criteria should be met: (a) identical indicators per measurement model, (b) identical data treatment, and (c) identical algorithm settings or optimization criteria^70^. In the present study, these requirements have been satisfactorily fulfilled. The second step of MICOM involves evaluating compositional invariance, which requires that the original correlations must be equal to or exceed the 5.00% quantile correlations^70^. Table 12 illustrates the results of compositional invariance, adhering to the aforementioned criteria.

**Table 12:** MICOM step 2 results report.

	Original correlation	Correlation permutation mean	5.00%	Permutation *p*-value
CI	1.000	1.000	1.000	0.008
CON	1.000	1.000	1.000	0.588
INQ	1.000	1.000	1.000	0.682
PU	1.000	1.000	1.000	0.497
SAT	1.000	1.000	1.000	0.661
SEQ	1.000	1.000	1.000	0.381
SYQ	1.000	1.000	1.000	0.56

The third step assesses the equality of mean values and variances across the composites within the groups. As articulated by Henseler et al.^70^, the difference in the original mean and variance should reside within the bounds of the 95% confidence interval. Tables 13 and 14 present the outcomes of testing for composite equality, thereby confirming the fulfillment of the aforementioned conditions. Upon establishing full measurement invariance, the composites from two distinct groups can be analyzed collectively, employing the pooled data.

**Table 13:** MICOM step 3 results report—mean.

	Original difference	Permutation mean difference	2.50%	97.50%	Permutation *p*-value
CI	0.281	−0.002	−0.238	0.323	0.119
PU	0.132	−0.001	−0.239	0.233	0.274
SAT	0.195	−0.002	−0.235	0.233	0.11

**Table 14:** MICOM step 3 results report—variances.

	Original difference	Permutation mean difference	2.50%	97.50%	Permutation *p*-value
CI	0.394	0.008	−0.428	0.448	0.209
PU	0.346	0.009	−0.479	0.513	0.103
SAT	0.335	0.009	−0.457	0.49	0.118

The concluding phase of the multi-group analysis necessitates identifying the existence of a substantial difference between the two groups. As recommended by Henseler et al.^70^, permutation p-values of 0.1 or less signify a statistically significant divergence between the groups. As represented in Table 15, the relationship between PU and SAT on CI does not exhibit a significant gender disparity. Therefore, research question 3 has been addressed.

**Table 15:** Permutation test path coefficient results.

	Original (male)	Original (female)	Original difference	Permutation mean difference	2.50%	97.50%	Permutation *p*-value
PU -> CI	0.397	0.42	−0.023	−0.002	−0.252	0.246	0.861
SAT -> CI	0.571	0.486	0.085	0.002	−0.244	0.257	0.514

### Common method variance

CMV denotes the systematic error arising from using a single measurement approach for the constructs under study^71^. This study employs two methods to gauge the prevalence of CMV. Initially, Harman’s single-factor analysis was conducted using the exploratory factor analysis approach, with all factor values standardized to a value of 1.0. The findings suggest CMV is not a substantial issue as only one factor emerged, explaining 37% of the variance in endogenous variables (which is less than the 50% threshold)^58^. Subsequently, a common method factor, encompassing all indicators of the primary constructs, was incorporated into the PLS model, allowing for the computation of each indicator’s variance substantively explained by the primary construct and the method^72^. As documented in the Appendix, the results indicate that the average substantively explained variance of the indicators is 0.890, with the average method-based variance being 0.022. The ratio of substantive variance to method variance is approximately 40:1. Furthermore, most method factor loadings are not statistically significant. Given the diminutive magnitude and lack of statistical significance of method variance, it is reasonable to conclude that CMV is unlikely to pose a significant concern in this study.

## Discussion

This study aims to investigate the factors that influence college students’ engagement with mobile learning. Drawing from a comprehensive review of existing literature, this research conjectures that factors such as INQ, SYQ, SEQ, CON, PU, and SAT play substantial roles in influencing a student’s CI to utilize mobile learning. The effectiveness of the proposed research model is evaluated using the PLS-SEM. A significant number of the postulated hypotheses are corroborated, explaining an impressive 85.1% of the total variance in the CI of college students to use mobile learning. A detailed discussion of the research findings in relation to the initially proposed research questions will follow in the subsequent sections.

College students’ PU of mobile learning has a significant positive influence on CI. This also aligns with our earlier observations, which demonstrated that PU is one of the most significant factors influencing the CI in blended learning for beginners^50^. A possible explanation for this is that mobile learning can meet the learning needs and goals of college students, thereby improving their academic performance. Additionally, mobile learning offers college students a user-friendly interface, comprehensive learning tools, and a seamless, personalized learning experience. Therefore, college students are more willing to continue using mobile learning.

College students’ SAT of mobile learning have a significant positive influence on CI. This finding is consistent with that of Shanshan and Wenfei^52^, who confirm that SAT is a vital predictor of CI based on the ECM, Task Technology Fit, flow theory, and trust in MOOCs. Several factors could explain this observation. Firstly, mobile learning can provide college students with a positive user experience, including well-designed interfaces and intuitive operation flows. Secondly, mobile learning can meet the learning needs of college students by providing high-quality learning resources, interactive learning tools, and personalized learning recommendations, thereby improving learning efficiency and achieving academic success. Ultimately, mobile learning can enable college students to learn anytime, anywhere, thereby enhancing the convenience and flexibility of their learning experience. As a result, the possibility of college students continuing to use mobile learning increases.

There was no significant gender difference between the PU of mobile learning and the CI of college students. This outcome is contrary to that of Nguyen et al.^56^, who found that gender has a significant moderating effect on the relationship between PU and CI. Several factors could explain this observation. On the one hand, the functions and features of mobile learning applications are designed to provide a convenient and useful learning experience for all learners, regardless of gender. On the other hand, more female college students are actively adopting mobile technology and applications than ever before. This trend may result in no significant difference in the PU of mobile learning applications among male and female college students.

There was no significant gender difference between the SAT of mobile learning and the CI of college students. This finding is consistent with that of Nguyen et al.^56^, who found that gender had no moderating effect on the relationship between SAT and CI. There are several possible explanations for this result. On the one hand, the relationship between college students’ SAT with mobile learning and CI may be related to the consistency of learning motivation, expectations, and goals. On the other hand, the relationship between the two constructs also depends on the user experience and learning efficiency of mobile learning. These factors are not directly related to gender.

INQ of mobile learning did not significantly affect college students’ CON. This outcome is contrary to that of Cheng^28^, who found that INQ contributed significantly to CON with the use of the blended e-learning system. This inconsistency may be due to the following reasons. On the one hand, the diversity of information sources in college students’ learning process, including classroom learning, books, and academic papers, weakens the impact of INQ on their CON in mobile learning. On the other hand, the information provided by mobile learning may not fully meet the individual needs of each student, thus reducing the dependence of college students on their information.

SYQ of mobile learning did not significantly affect college students’ PU. This finding is contrary to previous studies that have suggested SYQ is the most important factor affecting the PU of smartphone users^62^. These relationships may partly be explained by the fact that several factors, including the practicality of functions, the friendliness of interfaces, and the richness of learning resources influence the PU of college students to mobile learning. These factors may have a more significant effect on PU, thus weakening the effect of SYQ on PU.

SEQ of mobile learning did not significantly affect college students’ PU. However, this result has not previously been described^32^. The difference can be partly explained by the fact that college students’ PU may depend more on their existing experience and expectation setting of mobile learning rather than solely on the SEQ of mobile learning.

INQ of mobile learning has a significant positive influence on College students’ PU. A comparison of the findings with those of other studies confirms that INQ is an important antecedent of PU for smartphone users^62^. Several factors could explain this observation. Firstly, mobile learning that provides accurate, reliable, high-quality information can gain the trust of college students and improve their academic performance. Secondly, when the information presentation of mobile learning is easy to understand, and the content is diverse and rich, college students can more easily obtain and use learning information to improve Learning efficiency and achievement. Therefore, the PU of mobile learning for college students will be enhanced.

SYQ of mobile learning has a significant positive influence on College students’ CON. This finding is confirmed by Cheng^28^, who indicated that SYQ contributed significantly to CON in blended learning. The SYQ of mobile learning is reflected in the aspects of reliability, response speed, security, compatibility, user interface, etc. These factors can improve college students’ recognition and SAT of mobile learning and thus increase the degree of CON.

SEQ of mobile learning has a significant positive influence on College students’ CON. The result aligns with those of previous studies^28,29^. Based on an integrated model, Gu et al.^29^ confirmed that SEQ is one of the most crucial factors of CON in the MOOCs platform. A possible explanation for this might be that mobile learning can respond to users’ feedback and provide support in a timely manner, solve their problems and difficulties, and make users feel concerned and valued, thus improving the CON of mobile learning.

College students’ CON has a significant positive influence on the PU of mobile learning. This research finding is consistent with the results of Ashrafi et al.^37^, who proposed that students’ CON of the learning management system is an essential predictor of PU. There are several possible explanations for this result. Firstly, college students may have certain expectations for mobile learning, including convenient learning opportunities and high-quality educational resources. When they use these mobile learning tools and find that their expectations are met or exceeded, they are more likely to perceive them as useful. Secondly, the level of CON from college students regarding mobile learning is also likely to be influenced by user experience. If mobile learning offers a user-friendly interface, easy navigation, seamless interactions, and a satisfying learning experience, they are more inclined to have a positive CON of mobile learning, consequently perceiving it as a valuable tool.

College students’ CON of mobile learning has a significant positive influence on the SAT. This finding was also reported by Cheng et al.^41^, who demonstrated that expectation CON was the crucial predictor of the SAT during online collaborative learning. A possible explanation for this might be as follows. Firstly, mobile learning offers personalized configurations based on individual learning preferences and needs, catering to the diverse content and resource requirements of learners. This could maximize the CON of learners’ expectations, ensuring their needs are met to the greatest extent possible. Secondly, mobile learning allows college students to access learning materials and resources anytime and anywhere, effectively addressing their convenience demands.

College students’ PU of mobile learning has a significant positive influence on the SAT. This also aligns with our earlier observations, which demonstrated that PU has a significant impact on SAT scores for undergraduate and postgraduate students in India^45^. When college students perceive that mobile learning is beneficial to their studies, they are more motivated to utilize it and expect to achieve greater learning outcomes and a sense of fulfillment through their usage. The realization of this expectation will enhance college students’ SAT with mobile learning.

### Theoretical implications

The first contribution of this study is the development of a multi-dimensional comprehensive model for evaluating the CI of college students in mobile learning. Based on an in-depth review of the literature, the model integrates the ECM and quality factors (SYQ, INQ, and SEQ) of mobile learning. In other contexts, ECM and quality factors have been widely applied to investigate the CI of university students^29,73^. By examining the relationship between ECM, quality factors, and CI, this study confirms that they are also applicable to college students’ CI in mobile learning. The results indicate that ECM constructs and quality factors are significant predictors of college students’ CI when utilizing mobile learning.

The second contribution of this study concerns the explanatory and predictive power of the developed models. The model showed strong explanatory power in SAT and CI. The explanatory power of this model for the variance of college students’ SAT and CI with mobile learning is 87.8% and 85.1%, respectively. There has been a significant improvement compared to the previous model. In addition, this study evaluated the predictive power of the model using the RMSE method. The results indicated that the model had moderate predictive power. However, few existing studies have explored the predictive power of the model.

Finally, this study examines the moderating role of gender in the CI of university students towards mobile learning. Although previous studies have confirmed the moderating effect of gender in other contexts, this study found no significant moderating effect of gender on the relationship between PU, SAT, and CI towards mobile learning among university students.

### Practical implications

Research demonstrates that the quality of mobile learning has a significant effect on CI. Higher education institutions and teachers should ensure that the content is accurate, valuable, and aligns with the learning needs of college students. Developers should embed intelligent algorithms and learning analytics in mobile learning applications to provide students with learning suggestions and feedback based on their learning habits and progress, increasing their engagement and CI. Meanwhile, the learning content and functions should be updated regularly to maintain the freshness and attractiveness of mobile learning. Developers and information providers should provide regular training to service providers, enabling them to effectively address the challenges faced by college students during the learning process, actively respond to user suggestions and opinions, and enhance the functionality and user experience of the mobile learning system.

The study indicates that the relationship between PU, SAT, and CI of mobile learning among college students is not moderated by gender. Consequently, designers should focus on the user experience of college students when developing mobile learning, including usability, functionality, and interface design, to ensure that the mobile learning system meets their expectations and needs without requiring gender-specific adjustments or customization.

Based on ECM and quality factors, this study developed a CI model for mobile learning with high explanatory and predictive power. This provides a valid, reliable, and comprehensive analytical tool for universities and higher education institutions. This will greatly assist college students who adopt mobile learning in better understanding how to increase their SAT and CI scores using them.

### Limitations

Despite the significant findings of this study, some limitations and opportunities for future research remain. Firstly, while our research model mainly considers quality factors (INQ, SYQ, SEQ) and elements within ECM as factors influencing CI, there may be other potential factors that also impact the CI of university students towards mobile learning. For example, individual differences, learning styles, psychological characteristics, and situational aspects of the students might influence CI and could provide a more comprehensive perspective. Secondly, although this study has explored the moderating role of gender on the relationship between PU, SAT, and CI, future studies could consider other possible moderating variables, such as age, level of education, or experiences related to technology. Thirdly, the data for this study were collected from three universities in Jiangxi Province, China, limiting the generalizability of the findings to other cultural or geographic contexts. Future research might replicate this study in different cultural or learning contexts to further validate and generalize the findings. Finally, with the rapid development of artificial intelligence and big data technologies, future studies could explore how to integrate these emerging technologies into mobile learning to enhance their quality and impact on CI further. In summary, while this study provides a new theoretical model and empirical evidence for the CI of university students toward mobile learning, there remains a need for future research to delve deeper into both theoretical and methodological terms.

## Conclusions

The purpose of this study was to develop a comprehensive model to identify the factors influencing college students’ CI to use mobile learning. To achieve this goal, the study integrated quality factors (INQ, SYQ, and SEQ) with the ECM. Data were collected from three universities in Jiangxi Province, China, and analyzed using the PLS-SEM. The PU and SAT of university students using mobile learning systems have a significantly positive influence on their CI. Together, all antecedent variables account for 85.1% of the total variance in the CI of university students when using mobile learning systems. Furthermore, no significant gender differences were found in the impact of SAT and PU on CI. This study provides a unique perspective on college students’ CI to use mobile learning and may contribute to enhancing their engagement and satisfaction. Additionally, it offers an effective and reliable analytical tool for universities and higher education institutions.

## Supplementary Information

Below is the link to the electronic supplementary material.

Supplementary Material 1

## Data Availability

The data that support the findings of this study are available on request from the corresponding author.

## Abbreviations

CI: Continuance intention
PU: Perceived usefulness
SEQ: Service quality
INQ: Information quality
SYQ: System quality
SAT: Satisfaction
CON: Confirmation
TAM: Technology acceptance mode
ISSM: Information Systems Success Model
ECM: Expectation confirmation model
UTAUT: Unified Theory of Acceptance and Use of Technology
ECT: Expectation confirmation theory
MOOCs: Massive open online courses
PLS-SEM: Partial least squares structural equation model
VIF: Variance Inflation Factor
α: Cronbach’s alpha
R^2^: Explanatory power
f^2^: Effect size
Q^2^: Predictive power
CMV: Common method variance
AVE: Average variance extracted
MICOM: Measurement invariance of the composite model
