# Supplementary material for: Continuance intention to use mobile learning among college students: integrating quality factors and the expectation confirmation model
Source: Sci Rep. 2026 Jan 19;16:5691. doi: 10.1038/s41598-026-35949-w (PMC12891481; doi:10.1038/s41598-026-35949-w)
Supplement: Supplementary file 1 — Supplementary Material 1 [file 41598_2026_35949_MOESM1_ESM.doc]

Appendix A

|  | R1 | R1^2^ | R2 | R2^2^ |
| --- | --- | --- | --- | --- |
| Q9 | 0.952 | 0.906304 | 0.057 | 0.003249 |
| Q10 | 0.95 | 0.9025 | -0.08 | 0.0064 |
| Q11 | 0.95 | 0.9025 | 0.161 | 0.025921 |
| Q12 | 0.952 | 0.906304 | -0.016 | 0.000256 |
| Q13 | 0.953 | 0.908209 | 0.181 | 0.032761 |
| Q14 | 0.96 | 0.9216 | -0.18 | 0.0324 |
| Q15 | 0.908 | 0.824464 | 0.037 | 0.001369 |
| Q16 | 0.937 | 0.877969 | 0.221 | 0.048841 |
| Q17 | 0.965 | 0.931225 | -0.025 | 0.000625 |
| Q18 | 0.886 | 0.784996 | -0.181 | 0.032761 |
| Q19 | 0.939 | 0.881721 | -0.017 | 0.000289 |
| Q20 | 0.937 | 0.877969 | 0.058 | 0.003364 |
| Q21 | 0.95 | 0.9025 | -0.08 | 0.0064 |
| Q22 | 0.945 | 0.893025 | -0.047 | 0.002209 |
| Q23 | 0.952 | 0.906304 | -0.222 | 0.049284 |
| Q24 | 0.939 | 0.881721 | 0.031 | 0.000961 |
| Q25 | 0.939 | 0.881721 | 0.444 | 0.197136 |
| Q26 | 0.943 | 0.889249 | 0.1 | 0.01 |
| Q27 | 0.951 | 0.904401 | -0.099 | 0.009801 |
| Q28 | 0.957 | 0.915849 | -0.003 | 0.000009 |
| Q29 | 0.951 | 0.904401 | -0.109 | 0.011881 |
| Q30 | 0.931 | 0.866761 | -0.041 | 0.001681 |
| Q5 | 0.932 | 0.868624 | 0.058 | 0.003364 |
| Q6 | 0.953 | 0.908209 | 0.102 | 0.010404 |
| Q7 | 0.952 | 0.906304 | -0.091 | 0.008281 |
| Q8 | 0.939 | 0.881721 | -0.284 | 0.080656 |
| AVG |  | 0.889867346 |  | 0.022319346 |
| RATIO | 39.86977665 | | | |
